# Exome sequencing improves genetic diagnosis of congenital orofacial clefts

**DOI:** 10.3389/fgene.2023.1252823

**Published:** 2023-09-07

**Authors:** Shujuan Yan, Fang Fu, Ru Li, Qiuxia Yu, Fucheng Li, Hang Zhou, You Wang, Ruibin Huang, Chunling Ma, Fei Guo, Dan Wang, Xin Yang, Jin Han, Tingyin Lei, Dongzhi Li, Can Liao

**Affiliations:** Prenatal Diagnostic Center, Guangzhou Women and Children’s Medical Center, Guangzhou Medical University, Guangzhou, Guangdong, China

**Keywords:** genetic diagnosis, monogenic variants, congenital orofacial clefts, exome sequencing, cleft (lip and) palate

## Abstract

**Objective:** This retrospective study aims to evaluate the utility of exome sequencing (ES) in identifying genetic causes of congenital orofacial clefts (OFCs) in fetuses with or without other structural abnormalities, and to further explore congenital OFCs genetic causes.

**Methods:** The study enrolled 107 singleton pregnancies diagnosed with fetal OFCs between January 2016 and May 2022, and categorized them into two groups: isolated cleft lip and/or palate (CL/CP) and syndromic CL/CP. Cases with positive karyotyping and chromosomal microarray analysis results were excluded. Whole-exome sequencing was performed on eligible fetuses and their parents. Monogenic variants identified by ES and perinatal outcomes were recorded and evaluated during postnatal follow-up.

**Results:** Clinically significant variants were identified in 11.2% (12/107) of fetuses, with no significant difference in detection rate between the isolated CL/CP group and the syndromic CL/CP group (8/83, 9.6% vs. 4/24, 16.7%, *p* = 0.553). Additionally, sixteen (16/107, 15.0%) fetuses had variants of uncertain significance. We identified 12 clinically significant variations that correlated with clinical phenotypes in 11 genes from 12 fetuses, with *CHD7* being the most frequently implicated gene (n = 2). Furthermore, we observed a significant difference in termination rates and survival rates between the isolated CL/CP and syndromic CL/CP groups (41.0% vs. 70.8% and 56.6% vs. 20.8%, *p <* 0.05 for both).

**Conclusion:** Based on our findings, it is clear that ES provides a significant increase in diagnostic yield for the molecular diagnosis of congenital OFCs, thereby substantially improving the existing prenatal diagnostic capabilities. This study also sheds light on seven novel pathogenic variants, broadening our understanding of the genetic underpinnings of OFCs and expanding the disease spectrums of relevant genes.

## 1 Introduction

Congenital orofacial clefts (OFCs) are among the most common craniofacial birth defects worldwide, with an incidence of approximately 1/500 to 1/700 live births (Panamonta et al., 2015). OFCs are typically classified as non-syndromic cleft lip and/or cleft palate (NSCL/CP) or syndromic cleft lip and/or palate (SCL/CP) depending on whether cleft lip and palate in conjunction with other abnormal morphological characteristics or not (Luijsterburg and Vermeij-Keers, 2011; Shkoukani et al., 2013). The etiology of both NSCL/CP and SCL/CP is complex and influenced by both genetic and environmental factors. While the precise cause of congenital OFCs remains unknown, the significant role of genetics in the risk of the condition is widely recognized (Watkins et al., 2014; Beaty et al., 2016). In general, SCL/CP is often caused by variations or deletions in one or more genes, and is typically inherited in a Mendelian pattern. In contrast, NSCL/CP is more complex and is controlled by various genetic variants and risk factors. As a result, it does not follow Mendelian inheritance patterns (Leslie et al., 2016; Reynolds et al., 2020). Most OFCs without underlying genetic abnormalities can be corrected through surgical interventions after birth, with favorable outcomes (Dao and Goudy, 2016; Chou and Shih, 2020). Hence, accurate prenatal diagnosis of a genetic abnormality at the time of OFCs diagnosis is crucial for effective genetic counseling, pregnancy management, surveillance, and risk assessment.

Currently, karyotyping and chromosomal microarray analysis (CMA) are the most commonly used strategies for diagnosing OFCs during pregnancy. However, these methods are limited in their ability to detect chromosomal abnormalities, which account for only a fraction of all cases. In recent years, exome sequencing (ES) has revolutionized the field of genetics and become a powerful tool for genetic diagnosis in patients with suspected genetic disorders (Bamshad et al., 2011; Alazami et al., 2015). Although ES has become an essential tool in pediatric genetic diagnosis, it is not yet widely used in the prenatal setting. Recent studies have shown that ES has an added detection rate of 8.5%–10.3% for fetuses with suspected genetic disorders, compared to traditional diagnostic methods (Berk and Marazita, 2002; Wilhelm and Borgers, 2010). However, there have been relatively few studies that have employed ES to investigate the genetic causes of OFCs in the prenatal setting.

This study employed a retrospective analysis of pregnancies prenatally diagnosed with orofacial clefts (OFCs) by ultrasound, and excluded cases in which genetic testing via karyotyping or chromosomal microarray analysis (CMA) was positive. We then employed exome sequencing (ES) to further investigate the genetic causes and clinical outcomes in a cohort of 107 OFCs fetuses.

## 2 Materials and methods

### 2.1 Study participants

From January 2016 to May 2022, a total of 107 women with singleton pregnancies who had fetal OFCs detected by prenatal ultrasound were referred to our Prenatal Diagnostic Center for invasive prenatal diagnosis. The phenotype of all fetuses was performed by ultrasound examinations at our center to confirm the diagnosis based on the mid-sagittal, coronal and axial views of the fetal face and head (Wilhelm and Borgers, 2010). This study included fetuses diagnosed with OFCs through ultrasound was included with or without other structural abnormalities. After excluding cases with positive genetic testing results on karyotyping or chromosomal microarray analysis (CMA), the remaining cases without chromosomal abnormalities were offered the option of trio whole-exome sequencing. Informed consent was obtained from the pregnancies participating in this study before the invasive procedure.

### 2.2 Exome sequencing

Genomic DNA samples were extracted from chorionic villi, amniocytes, cord blood or parental blood using a Qiagen DNA Blood Midi/Mini kit (Qiagen GmbH, Hilden, Germany) following the manufacturer’s protocol. Whole-exome sequencing was provided in a familial trio after the exclusion of aneuploidy and pathogenic CNVs. Exome sequences were enriched by Agilent SureSelect human exome capture arrays (V6, Life Technologies, Carlsbad, CA, USA) and DNA libraries were prepared using a NEXTflex™ Rapid DNA Sequencing Kit (5144-02) (Bioo Scientific, Winchester, MA, USA) according to the manufacturer’s protocol. Samples were sequenced on a Hiseq XTen or Illumina Novaseq 6000 (Illumina, Inc., San Diego, CA). To generate raw data, we processed the raw image files using Bcl To Fastq (Illumina) for base calling. Trimmomatic was utilized to filter out low-quality and adapter contaminated reads with a quality score of ≥20 (Q20). The reads were aligned to the NCBI human reference genome (hg19/GRCh37) using BWA. Variant calling was accomplished using GATK and Picard, while SAMtools and Pindel were used for duplicate marking and recalibration, SNP analysis and indel realignment, respectively. All variants identified in each sample were annotated as either “novel” or “known” based on the SNP database (dbSNP). The minor allele frequencies (MAFs) of all known variants were annotated using various databases, such as dbSNP, the 1000 Genome Project, gnomAD, ExAC, EVS, and our local database. Gene and variants were annotated according to ClinGen, ClinVar, the professional version of the Human Gene Mutation Database (HGMD professional v2018.2 and v2021.2), previously associated diseases (based on Online Mendelian Inheritance in Man and Orphanet), and known functional domain data (according to Human Protein Reference Database and UniProtKB). The biological effects analysis of candidate variant genes was carried out using computational algorithms such as SIFT, PolyPhen2, MutationTaster, PROVEAN, Human Splicing Finder, MaxEntScan, and NNSplice. All selected variants were grouped into classifications of pathogenic, likely pathogenic, of uncertain significance (VUS), likely benign, or benign according to the ACMG guidelines. Sanger sequencing was performed to confirm all diagnostic genetic variants.

### 2.3 Modeling and interpretation of variant

For the CHD7 protein, the three-dimensional structure of the wild type and pathogenic variant type (c.4033C>T) were performed by SWISS-MODEL (http://swissmodel.expasy.org). The c.5535-2A>G was predicted by varSEAK (https://varseak.bio/index.php) and RDCC (https://rddc.tsinghua-gd.org/en/ai/rna-splicer).

### 2.4 Clinical follow-up

The pregnancy outcomes of our study were categorized as live birth, stillbirth, or termination. We performed a clinical follow-up assessment utilizing electronic medical records or telephone calls 6 months after the birth of the child, followed by routine annual follow-up assessments for all participants.

### 2.5 Statistical analyses

We carried out statistical analyses using SPSS software, version 26.0 (IBM). The chi-square test was used when the expected cell frequencies are reasonably large, typically greater than 5, and Fisher’s exact test was employed when the expected cell frequencies are small, typically less than 5, or when the sample size is small. The descriptive statistics were performed on means and ranges. Results were considered statistically significant when the *p*-value was less than 0.05.

## 3 Results

This retrospective study included 107 pregnancies diagnosed with congenital OFCs by ultrasound and who underwent invasive prenatal diagnostic procedures from January 2016 to May 2022. The mean age of the pregnant individual was 30.4 years (range 22-41), and the mean gestational age of the fetuses during the invasive procedure was 24.4 weeks (range 12-35). In those cases, there were 77 (72.0%) male fetuses and 30 (28.0%) female fetuses. The number of cleft lip and palate was significantly greater than that of only cleft lip and only cleft palate (81/107, 75.7% vs. 19/107, 17.8% vs. 7/107, 6.5%, *p <* 0.001). These cases were divided into two groups: with or without other structural deformities detected by ultrasound, known as isolated CL/P (n = 83) and syndromic CL/P (n = 24). Cardiac anomalies (11/24, 45.8%) and the skeletal system (6/24, 25.0%) were the most frequently associated structural anomalies. After the exclusion of aneuploidies and pathogenic CNVs by karyotyping and CMA, there were 107 cases were subjected to ES. The additional detection rate with ES was 11.2% (12/107), including five cases (5/107, 4.7%) with pathogenic genetic variants and seven cases (7/107, 6.5%) with likely pathogenic genetic variants. However, the detection rate of pathogenic and likely pathogenic genetic variants did not significantly differ between the isolated SCL/CP group and the syndromic CL/CP group (8/83, 9.6% vs. 4/24, 16.7%, *p* = 0.553). Moreover, sixteen cases had variants of uncertain significance (VUS) (Supplementary Table S1).

We detected 12 clinically significant variants from 11 genes in 12 fetuses with congenital OFCs. These genes included *CHD7* (n = 2)*, KMT2D* (n = 1)*, FLNB* (n = 1)*, EYA1* (n = 1)*, COL2A1* (n = 1)*, ARHGAP29* (n = 1)*, GLI2* (n = 1)*, COL11A1* (n = 1)*, MID1* (n = 1)*, FGFR2* (n = 1)*, FLNA* (n = 1)*.* All observed variants were linked to congenital OFCs, and seven novel variants were first reported, which expanded the variation spectrums of OFCs-related genes. Out of these 12 detected variants, nine (75.0%) were *de novo* variants that followed autosomal dominant inheritance (six missense variants, two truncating variants, and one splice variant), while one missense variant was *de novo* following X-linked dominant inheritance. Four of the variants were involved with craniofacial morphogenesis, including (*COL2A1*_i004): p.(Glu688Ter), (*COL11A1*_i008): p.(Gly1150Val), (*FGFR2*_i0011): p.(Ser252Trp), (*GLI2*_i006): p.(Tyr296Ter), which resulted in abnormalities such as Stickler syndrome, type 1 (OMIM #120140), Marshall syndrome (OMIM #154780), Apert syndrome (OMIM #101200), Culler-Jones syndrome (OMIM #165230), etc. Four other variants were involved in facial features, nervous and cardiovascular systems, including (*KMT2D*_i001): p.(Arg5225Cys), (*CHD7*_i007): p.(Arg1345Cys), (*CHD7*_i009): p.(?), (*MID1*_i010): p.(His600ProfsTer12). Two variants, (*FLNB*_i002): p.(Pro2020Leu), (*FLNA*_i012): p.(Gly1554Arg)*,* were involved in filamins, which are associated with cell-ECM adhesion and resulted in musculoskeletal abnormalities. Notably, despite both (*ARHGAP29*_i005): p.(?) and (*EYA1*_i003): p.(Arg106Ter) being found in isolated CLP cases on prenatal ultrasound, *ARHGAP29* was more commonly associated with NSCL/CP, while *EYA1* could cause hearing impairment or kidney abnormalities (OMIM #602588). In addition, three (25%) fetuses had inherited the relevant variations: cases 5 and 6 both had autosomal dominant variations inherited from their affected mother and no affected father respectively. Case 10 detected a hemizygous variant in *MID1*, which was an X-linked recessive inheritance mode. The genetic testing results are presented in Table 1.

**TABLE 1:** Details of pathogenic (P) or likely pathogenic (LP) sequence variants of OFCs fetuses.

Case	Ultrasound findings	Gene	References sequence	Nucleotide/protein position	Whether gnomAD and in-house database documented?	pathogenic variant type and classification	Disease (OMIM ID)	Outcome
1	Cleft palate	*KMT2D*	NM_003482.3	c.15673C>T^a^	NO/NO	Het, *De novo*, AD, P	KABUK1 (147920)	TOP
				p.(Arg5225Cys)				
2	Cleft lip	*FLNB*	NM_001164317.2	c.6059C>T^a^	NO/NO	Het, *De novo*, AD, LP	LRS (150250)	TOP
				p.(Pro2020Leu)				
3	Cleft lip and palate	*EYA1*	NM_001370333.1	c.316C>T^a^	NO/NO	Het, *De novo*, AD, P	BOS1 (602588)	Live birth
				p.(Arg106Ter)				
4	Cleft lip and palate	*COL2A1*	NM_001844.5	c.2062G>T^a^	NO/NO	Het, *De novo*, AD, P	STL1 (108300)	TOP
				p.(Glu688Ter)				
5	Cleft lip and palate	*ARHGAP29*	NM_001328664.2	c.1920 + 1G>A	NO/NO	Het, Mat(affected), AD, LP	(610496)	TOP
				p.(?)				
6	Cleft lip and palate	*GLI2*	NM_005270.5	c.887dup^a^	NO/NO	Het, Pat, AD, P	CJS (615849), HPE9 (610829)	TOP
				p.(Tyr296Ter)				
7	Cleft lip and palate	*CHD7*	NM_017780.3	c.4033C>T	NO/NO	Het, *De novo*, AD, LP	Charge syndrome (214800), KS (612370)	TOP
				p.(Arg1345Cys)				
8	Cleft palate; micrognathia; bilateral subependymal cyst; polyhydramnios	*COL11A1*	NM_080629.3	c.3449G>T^a^	NO/NO	Het, *De novo*, AD, LP	STL2 (604841), MRSHS (154780)	Live birth
				p.(Gly1150Val)				
9	Cleft lip and palate; cervical lymphatic hygroma	*CHD7*	NM_017780.3	c.5535-2A>G^a^	NO/NO	Het, *De novo*, AD, LP	Charge syndrome (214800), KS (612370)	Embryonic death
				p.(?)				
10	Cleft lip and palate; persistent left superior vena cava; widened posterior cranial fossa	*MID1*	NM_033290.3	c.1798dup	NO/NO	Het, Mat, XLR, LP	GBBB (300000)	TOP
				p.(His600ProfsTer12)				
11	Cleft palate; craniosynostosis; syndactyly of hands and feet	*FGFR2*	NM_000141.5	c.755C>G	NO/NO	Het, *De novo*, AD, P	Apert syndrome (101200)	TOP
				p.(Ser252Trp)				
12	Cleft lip and palate; bilateral multicystic dysplastic kidneys	*FLNA*	NM_001110556.2	c.4660G>A	NO/NO	Het, *De novo*, XLD, LP	FGS2 (300321); CVDPX (314400); OPD1 (311300)	TOP
				p.(Gly1554Arg)				

OFCs, Orofacial clefts; Het, Heterozygous; AD, autosomal dominant; TOP, termination of pregnancy; Mat, Maternal inherited; XLR, X-linked recessive; XLD, X-linked dominant inheritance; KABUK1, Kabuki syndrome 1; LRS, larsen syndrome; MRSHS, marshall syndrome; BOS1, Branchiootorenal syndrome 1; AOS1, Adams-oliver syndrome 1; STL1, Stickler syndrome 1; CJS, Culler-Jones syndrome; HPE9, Holoprosencephaly 9; KS, kallmann syndrome; GBBB: Opitz G/BBB, syndrome; FGS 2, FG, syndrome 2; CVDPX, cardiac valvular dysplasia, X-linked; OPD1, otopalatodigital syndrome, type I.

^a^ The frst reported variant.

**FIGURE 1:**
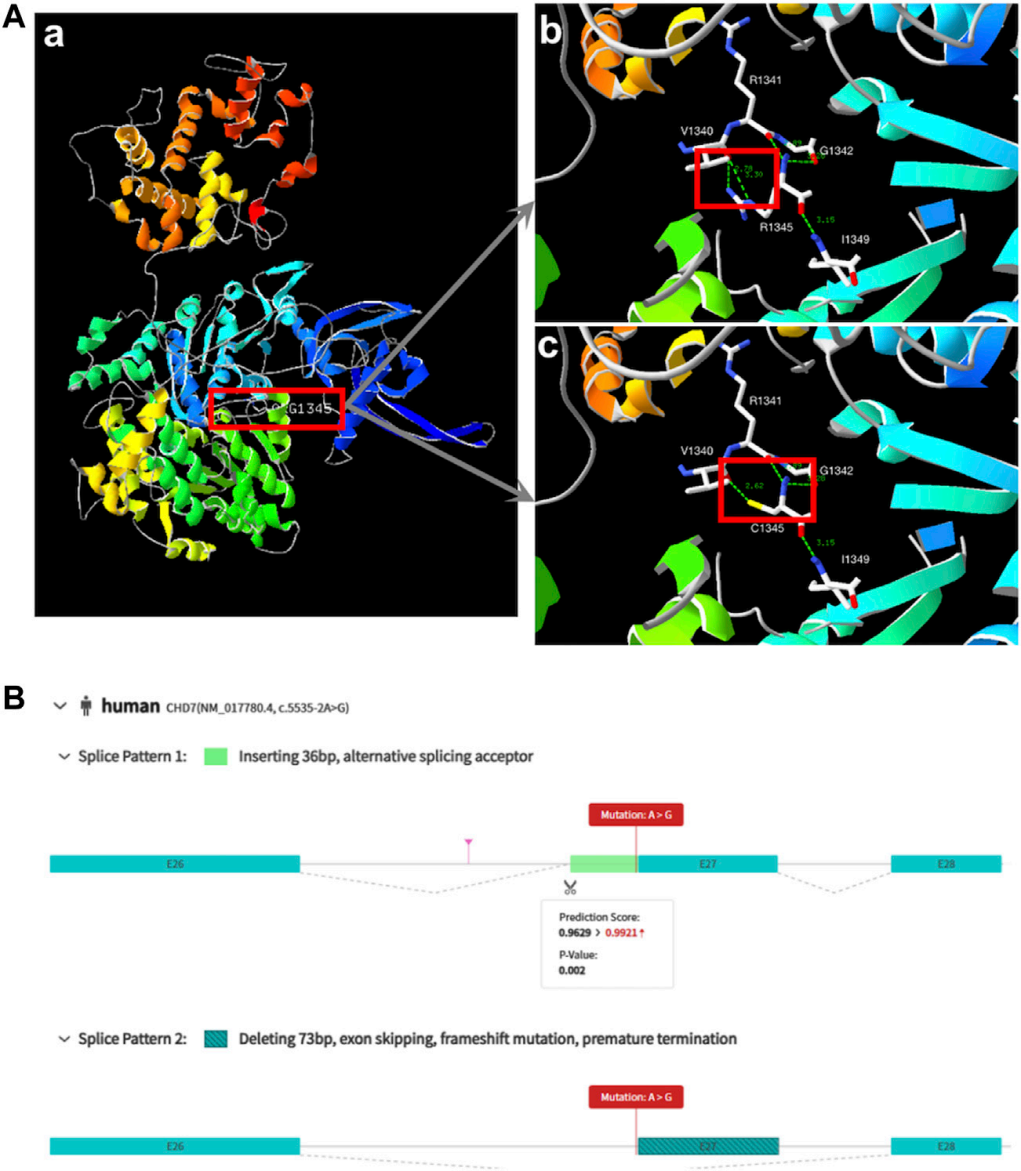
Biophysical analyses of the *CHD7*: **(A)** (a/b) Part of the three-dimensional structure of the wild type *CHD7*; (c) pathogenic variant type (c.4033C>T; p.Arg1345Cys). **(B)** The prediction of two splice patterns of *CHD7* (c.5535-2A>G).

Based on predictive analysis of three-dimensional protein structure of the *CHD7*, it has been showed that Arg1341, Val1340, Gly1342 And Ile1349 were linked by hydrogen bonds in the wild type. After the variation the hydrogen bonds between Arg1341 and Val1340 disappeared (Figure 1A). ES analysis uncovered a splice site variant (c.5535-2A>G) within the *CHD7* gene. Computational predictions by varSEAK (https://varseak.bio/index.php) and RDCC (https://rddc.tsinghua-gd.org/en/ai/rna-splicer) suggest that this variant exerts an impact on RNA splicing result in two distinct patterns. The first pattern involves the insertion of a 36-base pair sequence between exon26 and exon27, potentially leading to alternative splicing acceptor sites. The second pattern entails a deletion of 73 base pairs, resulting in exon 27 skipping, and premature termination (Figure 1B).

Out of the 107 pregnancies included in our study, pregnancy outcomes were obtained in 105 (98.1%) cases, 51 (47.7%) families chose to terminate the pregnancy, 52 (48.6%) were livebirth, one (0.9%) newborns died after birth, one (0.9%) fetal demised intrauterine and other two (1.9%) cases were lost to follow-up. Table 2 summarizes the perinatal outcomes of our cohort. Notably, a positive family history of CL/P was found in 16.8% (18/107) of these families, with a higher percentage of affected relatives observed in the isolated CL/P group than in the syndromic CL/P group (16/18, 88.9% vs. 2/18, 11.1%). We found a significant difference in termination rates and surviving rates between the isolated CL/P and syndromic CL/P (41.0% vs. 70.8% and 56.6% vs. 20.8%, *p* = 0.010 and *p* = 0.002). Furthermore, 42 fetuses had received surgical repair: 38 (73.1%) enjoyed good outcomes, and four (7.7%) possessed other major structural abnormalities required additional intervention with or without developmental delay. The other ten (19.2%) were waiting for an opportunity for operation. Overall, the repair operation rates for CL/P were observed to be 80.9% (38/47) in the isolated group and 80.0% (4/5) in the syndromic group.

**TABLE 2:** The perinatal outcomes in fetuses with OFCs.

		Isolated CL/P vs. SCL/P			CL vs. CP vs. CLP				Genetic testing results			
Perinatal outcome	Total (n = 107)	Isolated CL/P (n = 83)	SCL/P (n = 24)	*p*-value	CL only (n = 19)	CP only (n = 7)	CLP (n = 81)	*p*-value	CSVs (n = 12)	VOUS (n = 16)	Negative (n = 79)	*p*-value
TOP	51(47.7%)	34(41.0%)	17(70.8%)	0.010^a^	4(21.1%)	4(57.1%)	43(53.1%)	0.037^b^	9(75.0%)	9(56.3%)	36(45.6%)	0.145^a^
Live birth	52(48.6%)	47(56.6%)	5(20.8%)	0.002^a^	15(78.9%)	2(28.6%)	35(43.2%)	0.011^b^	2(16.7%)	7(43.8%)	32(40.5%)	0.254^b^
Embryonic death	1(0.9%)	0	1(4.2%)	/	0	0	1(1.2%)	/	1(8.3%)	0	0	/
Neonatal death	1(0.9%)	0	1(4.2%)	/	0	1(14.3%)	0	/	0	0	0	/
Lost to follow up	2(1.9%)	2(2.4%)	0	/	0	0	0	/	0	0	0	/

^a^ The chi-square test is employed in this analysis.

^b^ The Fisher’s exact test is employed in this analysis; OFCs, Orofacial clefts; CLP, Cleft lip or/and palate; CSVs, clinically significant variants; VUS, variants of unknown significance; TOP, termination of pregnancy.

## 4 Discussion

Orofacial clefts (OFCs) are the most prevalent congenital craniofacial anomalies globally and pose a significant burden on affected individuals and their families (Berk and Marazita, 2002). While the etiology of OFCs remains incompletely understood, most studies recognize the critical role of genetic factors in craniofacial dysplasia (Beaty et al., 2016; Saleem et al., 2019). Although genetic elements are implicated, routine diagnostic tools for CL/P include karyotyping and CMA, which are capable of detecting chromosomal abnormalities but not single nucleotide variations (SNVs). The widespread implementation of ES in the prenatal setting has provided additional insight into the genetic factors responsible for CL/P, leading to the identification of novel copy number variations (CNVs) in known genes and risk loci associated with CL/P, including the rs145192286 variant in LAMA5, region 1p33-36 in GRHL3 and IRF6 (Basha et al., 2018; Fu et al., 2023). Our prior research showed detection rates of 3.6% (5/139) for abnormal karyotypes and 7.7% (11/143) for pathogenic CNVs in prenatal genetic diagnosis of fetal CL/P using karyotype analysis and CMA, respectively (Jin et al., 2018). However, there is limited research exploring the genetic and clinical value of ES in congenital OFCs. This study aims to further investigate the underlying genetic etiology and clinical outcomes of a cohort of pregnancies complicated by congenital OFCs using ES.

Our study employed ES to perform genetic analysis on 107 pregnancies, all of whom had been prenatally diagnosed with congenital OFCs via ultrasound and received negative results on both karyotype testing and CMA. Pathogenic or likely pathogenic variants were identified in 11.2% (12/107) of pregnancies with fetal OFCs, indicating that ES provides a high additional diagnostic yield for molecular diagnosis of congenital OFCs. In the investigated cohort, there was no significant difference in detection rate between the isolated cleft lip and palate (CL/P) group and the syndromic CL/P group (8/83, 9.6% vs. 4/24, 16.7%, *p* = 0.553), indicating that an efficient tool can accurately identify specific variants and candidate gene loci in the etiology of both isolated and syndromic OFCs. Additionally, this study uncovered seven novel pathogenic variants, broadening the spectrum of OFC-related genes implicated in this condition.

In the syndromic CL/P group evaluated in our study, the most commonly observed structural malformations were those of the cardiovascular and skeletal systems, in agreement with previous research (Impellizzeri et al., 2019). Among these co-occurring cardiac anomalies, the most frequent were those combined with atrioventricular septal defect (6/11, 54.5%), followed by pulmonary valve stenosis or atresia (4/11, 36.4%). It is important to note that six of the eleven identified genetic variants were implicated not only in CL/P but also in other phenotypes of cardiac abnormalities. The co-occurrence patterns of clefts and cardiovascular defects may be related to the mechanisms of early embryonic development, during which the aortic arches of the primitive heart surround the pharyngeal arches that eventually give rise to the face (Genisca et al., 2009). Consequently, we recommend that clinicians conduct enhanced cardiac ultrasound monitoring in fetuses when fetal CL/P is detected by ultrasound. Furthermore, it is worth noting that skeletal or neurodevelopmental conditions are more likely to co-occur with CL/P. Our study revealed that variants in six (54.5%) of the eleven identified genes may result in skeletal abnormalities, including *FLNB*, *COL2A1*, *COL11A1*, *FGFR2*, *FLNA*, and *GLI2*. Case 11 showed prenatal ultrasound evidence of craniosynostosis and syndactyly of hands and feet, while case 8 demonstrated prenatal ultrasound evidence of micrognathia and bilateral subependymal cysts. Therefore, serial ultrasound assessments of fetuses with CL/P are important to rule out CL/P as the initial sign of skeletal abnormalities or central nervous system disorders.

In our study, *CHD7* was the most frequently identified gene (n = 2). *CHD7* plays a critical role in the formation of a multipotent migratory neural crest, which affects craniofacial bones and cartilages, the peripheral nervous system, pigmentation, and cardiac structures (Bajpai et al., 2010). Variations in the *CHD7* gene are linked to various developmental disorders, including CHARGE syndrome (OMIM #214800) and Kallmann syndrome. CHARGE syndrome is characterized by a cluster of congenital anomalies such as choanal atresia, and malformations of the heart, inner ear, and retina, while Kallmann syndrome (KS) is an inherited developmental disease that combines congenital hypogonadotropic hypogonadism with anosmia. Cleft lip and palate may be present in both of these syndromes (Källén et al., 1999; Marcos et al., 2014). Case 7 was diagnosed with CLP by prenatal ultrasound, and a missense variant (c.4033C>T; p.(Arg1345Cys)) in the *CHD7* gene was identified, which had been listed in the dbSNP database with the ID rs1563644113. This sequence alteration replaced arginine, which is basic and polar, with cysteine, which is neutral and slightly polar, at codon 1345 of the *CHD7* protein. Bioinformatics software predicted that this variant would result in the disruption of the *CHD7* protein function. Case 9 exhibited cervical lymphatic hygroma and CLP by first-trimester ultrasound in pregnancy. Chorionic villus sampling was conducted, but the pregnancy was lost before the results were obtained. A splice site variation (c.5535-2A>G) was identified in this gene. This variant is the first reported variant of *CHD7* and bioinformatics software predicted that this variant would lead to protein truncation and an early termination codon. Figure 1 displays the variant locations in the protein. Because both of the variants identified in our study were *de novo* and caused syndromes that were severe but not rare, we suggest that the analysis of *CHD7* gene variants should be included in the prenatal diagnosis of Chinese pregnancies associated with suspected CL/P. Furthermore, it is necessary to conduct a trio-ES examination that includes the proband rather than just a carrier screening panel for parents.

Variations in genes encoding cartilage collagens II and XI, *COL2A1* and *COL11A1*, have been associated with chondrodysplasias, which are often associated with Robin sequence, micrognathia, or cleft palate (Melkoniemi et al., 2003). In our study, case 4 had a nonsense variant identified in c.2062G>T of *COL2A1*, leading to early truncation of the *COL2A1* protein (Glu688 to stop, Glu688Ter). One-third of all variants are dominant-negative variants that affect the glycine residue in the G-X-Y repeats sequences, and such variants resulting in a premature stop codon are usually associated with less severe phenotypes such as Stickler syndrome (Barat‐Houari et al., 2016). They may lead to various abnormalities in the ocular, orofacial, skeletal and audiological systems. Despite this, case 4 only presented with CLP, and no other skeletal anomalies were observed. In contrast, characteristics of the Marshall-Stickler syndrome spectrum caused by *COL11A1* differ from those caused by *COL2A1*, with patients harboring *COL11A1* variants exhibiting more severe hearing impairment but rare vitreoretinal degeneration and retinal detachments compared to those with *COL2A1* variants (Annunen et al., 1999). Case 8, with prenatal ultrasound detecting cleft palate, micrognathia, polyhydramnios, and bilateral subependymal cysts, was found to harbor a missense variant (c.3449G>T; p.(Gly1150Val)) in *COL11A1*, further highlighting the phenotypic overlap and extensive phenotypic variation occurring in collagenopathies caused by *COL2A1* and *COL11A1*. Additionally, these two novel described variations were the first reported, and served to expand the variation spectrum associated with the *COL2A1* and *COL11A1* genes.

**FIGURE 2:**
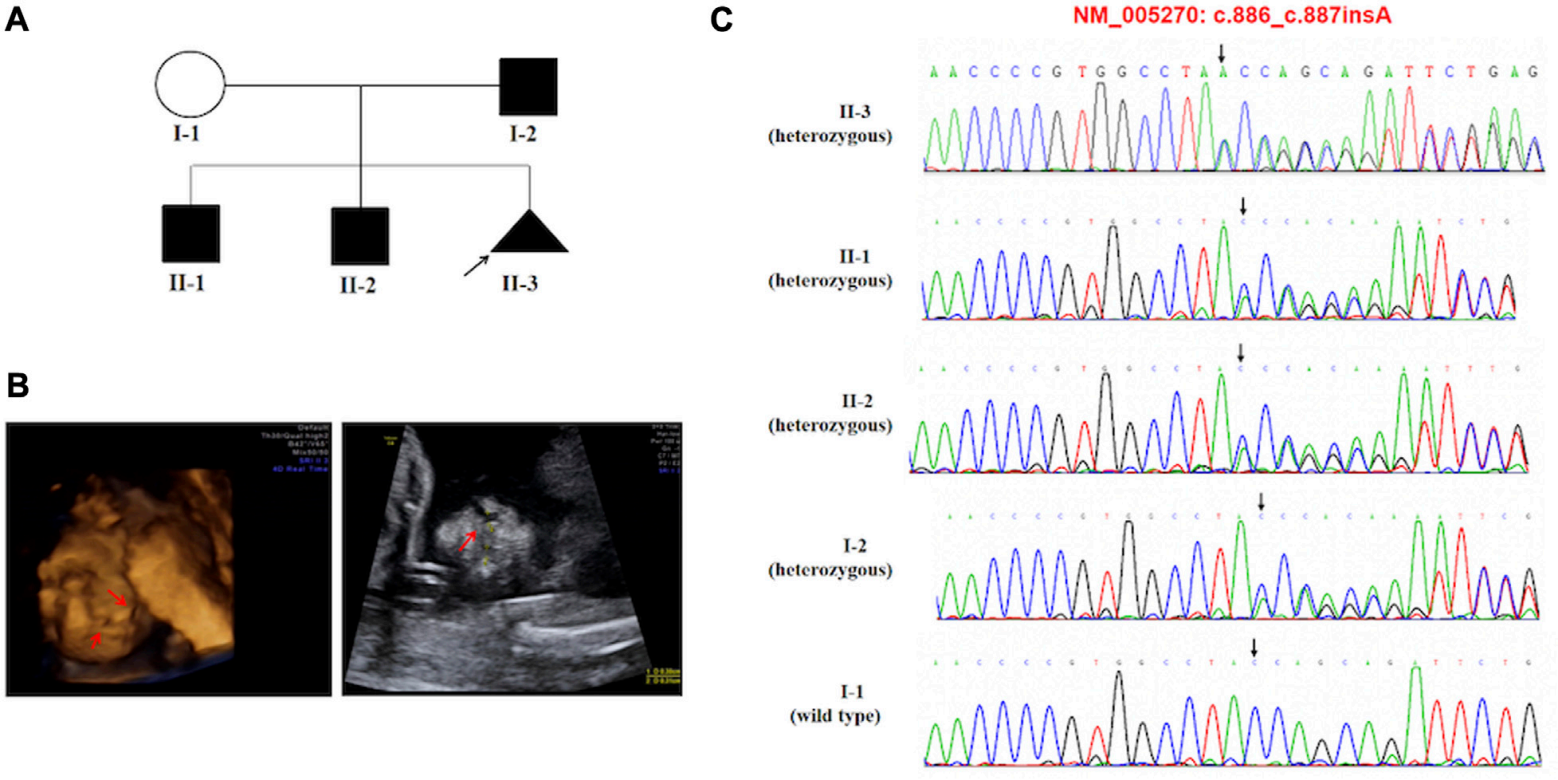
**(A)** Pedigree of the family carried the c.887dup (p.Tyr296Ter) pathogenic variant of *GLI2*. **(B)** Ultrasonographic findings of the cleft and palate in the fetus (II-3). **(C)** Sequencing result of the *GLI2* pathogenic variant. Sequence chromatogram indicates a heterozygous pathogenic variant c.887dup (p.Tyr296Ter).

Activating variants in the extracellular domains of *FGFR1-3* are responsible for numerous human skeletal disorders, including various craniosynostosis syndromes (Wilkie et al., 1995). In our study, we identified a variant in *FGFR2*, c.755C>G (p.(Ser252Trp)), in case 11, which is associated with the development of Apert syndrome (OMIM #101200). Apert syndrome is a rare genetic disorder characterized by an array of clinical features, including craniosynostosis, midface retrusion, and limb anomalies (Willie et al., 2022). A cleft palate is present in a subset of Apert syndrome patients. Over 98% of Apert syndrome cases are attributed to two amino acid substitutions, Ser252Trp (S252W) and Pro253Arg (P253R), which are located in the linker region between the second and third extracellular Ig domains of *FGFR2* (Park et al., 1995; Wilkie et al., 1995). Additionally, *GLI2* has been shown to act downstream of *FGFR2*. We identified a novel frameshift variant in c.887dup p.(Tyr296Ter). of *GLI2* in case 6. *GLI2*, *SHH*, and *PTCH1* represent key hedgehog pathway members, which are critical for craniofacial patterning, and variations in these genes have been associated with holoprosencephaly with CL/P (Roessler et al., 1996; Ribeiro et al., 2006). The *GLI2* gene has been linked to Culler-Jones syndrome and Holoprosencephaly 9 (HPE9), and studies have reported that patients with pathogenic variants of the *GLI2* gene demonstrate an autosomal dominant inheritance pattern, variable expressivity and incomplete penetrance (Elward et al., 2020). Bertolacini *et al.* reported the identification of a heterozygous 2-bp deletion (864delCC) in the *GLI2* gene in a Brazilian family exhibiting variable manifestations of HPE9 (Bertolacini et al., 2012). The frameshift resulted in a premature termination p.(Pro288fsTer301). The variant was inherited from the mother, who had postaxial polydactyly. The affected child exhibited microcephaly, a large cleft lip/palate involving partially the premaxilla, and bilateral postaxial polydactyly. At 8 years of age, the child showed severe neurodevelopmental delay and brain magnetic resonance imaging (MRI) revealed semi lobar holoprosencephaly. Case 6 is noteworthy as the variant was inherited from their father (I-2), who exhibited normal phenotypes. The index proband (II-3) was a 16-gestational-week fetus with CLP indicated by prenatal ultrasound screening. This family has two boys (ages 10 and 8) with different degrees of language development delay and intellectual disability. The 10-year-old boy (II-1), with mild symptoms with an evaluation score of 53 by the Wechsler Intelligence Scales, and the 8-year-old boy (II-2) presented with moderate language development delay and intellectual disability, obtaining an evaluation score of 47 by the Wechsler Intelligence Scales. Furthermore, the 8-year-old boy (II-2) was diagnosed with congenital hypospadias at birth, and seizures began at age 5. Brain MRI showed cystic posterior fossa anomalies and cerebellar dysplasia. Sanger sequencing results demonstrated that all of the children in this family inherited the variation (c.887dup p.(Tyr296Ter)). in the *GLI2* gene, a variant first reported and predicted by bioinformatics software to disrupt *GLI2* protein function. Most known pathogenic variants of this gene are truncated variants, including frameshift and nonsense variants. The variant was not indexed in the dbSNP, gnomAD, or 1000 Genomes databases, and based on the ACMG classification criteria, it was considered pathogenic. The frameshift variant observed in the *GLI2* gene resulted in highly variable clinical expressions ranging from minor facial signs to complex nervous system anomalies in this family. The ultrasonography findings and the details associated with the identification of these variations in this family are depicted in Figure 2.

Within our study, eighteen families (16.8%) exhibited a positive family history of CL/CP; of these, two cases (cases 5 and 10) were attributed to pathogenic variants. Figueiredo *et al.* found a robust association between OFCs and family history, suggesting that it is a crucial factor to consider when predicting the potential risk of new cases of OFCs within a family (Figueiredo et al., 2015). In case 5, we detected fetal isolated CLP via ultrasound examination, after which genetic testing revealed a splice site variant (c.1920 + 1G>A) in the *ARHGAP29* gene. Further investigation of the family lineage revealed that the affected mother and grandfather harbored the same pathogenic variant, which conformed to an autosomal dominant mode of inheritance and was consistent with a non-syndromic form of CL/CP (Yu et al., 2022). This precise diagnosis provided the family with confidence, enabling them to continue with the pregnancy. The baby was born with a confirmed diagnosis and underwent surgical repair after birth with a favorable outcome. In the case of case 10, we observed fetal CLP with cystic hygroma, persistent left superior vena cava, absence of nasal bone, and Blake cyst via ultrasound examination at 17 gestational weeks in two consecutive pregnancies. Genetic diagnosis via amniocentesis was performed, revealing that the fetus carried the c.1798dup p.(His600ProfsTer12) pathogenic variant of *MID1*. The pathogenic variant was inherited from the mother with mild phenotypes, including congenital heart disease (ventricular deficiency and pulmonary stenosis). We hypothesized that the previously affected pregnancy was also caused by pathogenic variants in this gene. The results of this prenatal diagnosis can serve as a valuable guide for the couple in their future pregnancy planning. The pathogenic variant identified in this case has been reported in several individuals with X-linked Opitz GBBB syndrome (OMIM #300000) (Cho et al., 2006), and is included in the HGMD database under ID CI067580. The MID1 gene encodes a microtubule-associated RING-Bbox-Coiled-coil (RBCC) protein and Cainarca *et al.* suggest that midin is involved in the formation of multiprotein structures and that impaired association with these cytoskeletal structures causes the developmental defects of Opitz syndrome (Cainarca et al., 1999). Previous studies have shown that in addition to *MID1*, genes encoding components of cytoskeletal proteins such as *FLNA*, *VIL1*, and *FLNB* play a role in the development of CLP. Overall, we suggest that variants in genes encoding cytoskeletal proteins during development are deemed crucial to CLP formation. Opitz GBBB is a congenital midline malformation syndrome characterized by hypertelorism, CL/P, laryngo-tracheo-esophageal abnormalities (So et al., 2005), genito-urinary abnormalities, developmental delay, and cardiac defects. However, heterozygous females may exhibit mild features of Opitz GBBB, while clinical presentations in affected males are quite variable (De Falco et al., 2003). As CLP is the most common facial abnormality characterizing Opitz GBBB with an incidence of around 50% in males with identified *MID1* variants (De Falco et al., 2003; Li et al., 2016), and diagnosis of CLP in the first trimester is feasible, we suggest prenatal diagnosis of Opitz GBBB in fetuses with CL/P and a history of family disease.

It is necessary to conduct postnatal follow-ups for children with a prenatal diagnosis of CL/P. Our study collected data on pregnancy outcomes and postpartum treatment of live birth cases. Based on our findings, isolated CL/P had a better prenatal and postnatal prognosis than syndromic CL/P, and fetal CL/P associated with other anomalies correlated with an increased rate of pregnancy termination. Of the 52 live birth cases, 73.1% (40/52) had successful postnatal surgical repairs and normal development without major structural defects, while 7.7% (4/52) had other significant structural abnormalities that required additional intervention. Two of these cases exhibited pathogenic variants, one case presenting a heart defect (ventricular septal defect and aberrant right subclavian artery), and the other case showing sensorineural hearing loss. The majority of children generally experience good health and normal neurodevelopment, excluding genetic factors according to previous literature. *Burnell et al.* reported a live birth rate of 84.5% (82/97) and 84.4% (65/77) long-term favorable outcomes without complex disorders in isolated CL/P cases. Li *et al.* found a live birth rate of 43.9% (29/66) and 87.5% (21/24) had good long-term outcomes without any detectable pathogenic genetic abnormalities (Burnell et al., 2014; Li et al., 2022). In our study, the live birth rate was 48.6% (52/107), similar to Li’s study. This feature may be explained by a selection bias, in which the cases in both studies included isolated CL/P and syndromic CL/P. However, our findings indicated that all cases were subjected to CMA and ES to exclude chromosomal abnormalities and monogenic diseases. Nevertheless, the detection rate was not significantly different between isolated CL/P and syndromic CL/P (8/83, 9.6% vs. 4/24, 16.7%, *p* = 0.553). We suggest that expecting mothers with a fetus diagnosed with OFCs, whether isolated or syndromic CL/P, be offered ES as a part of their prenatal testing, particularly in cases where no abnormalities have been identified through karyotyping and CMA. In summary, a definitive genetic diagnosis and a positive prognosis provide reassurance to expecting mothers and increase the likelihood of carrying the pregnancy to term, especially for those carrying fetuses with isolated CL/P having received negative results. Providing this information enables expecting parents to make informed decisions with confidence in proceeding with their pregnancy.

The study presents several limitations. Firstly, the retrospective study design employed in this investigation may have led to recall bias, thus impacting the quality of the data collected from the study participants. Based on the available data and the design of the study, it is important to acknowledge that utilizing these findings as the sole justification for additional genetic testing and enhancing specificity in the diagnosis of OFCs presents a limitation. Secondly, it is important to consider that while ES is a phenotype-driven detection technique, prenatal ultrasound imaging provides limited information on fetal phenotypes. That primarily focuses on assessing the structural development of the fetus and the gestational age can affect the visibility of certain structures and anomalies during ultrasound. On the one hand, This may impact our ability to determine whether CL/CP are associated with other malformations. On the other hand, this may compromise the data filtering and analysis process, potentially resulting in the inadvertent omission of crucial information. Finally, it is worth noting that the ES protocol utilized in the current study only focused on the protein-coding exonic regions and some adjacent intronic regions, thus neglecting non-coding regions and epigenetic exertions as potential contributing factors to the development of congenital orofacial clefts. However, it should be acknowledged that these elements can play a significant role in the occurrence of this condition and therefore should be assessed in future research.

## 5 Conclusion

To date, this is the largest prenatal study to employ ES for the genetic analysis of fetal OFCs cases. In light of our findings, it appears that ES has a notably high rate of improving the overall diagnostic yield for molecular diagnosis of fetal orofacial clefts. Therefore, we strongly advocate for the implementation of ES in clinical practice for pregnancies diagnosed with fetal OFCs, in cases where conventional karyotyping and CMA have produced negative results. Furthermore, this study has resulted in the discovery of seven novel pathogenic variants, thus expanding the understanding surrounding genes associated with congenital OFCs and further contributing to the clinical knowledge base in this field.

## Data Availability

The data supporting the findings of this study are not publicly available due to privacy concerns for the research participants. Further inquiries regarding the data can be directed to the corresponding author.

## References

[B1] Alazami A. M. Patel N. Shamseldin H. E. Anazi S. Al-Dosari M. S. Alzahrani F. (2015). Accelerating novel candidate gene discovery in neurogenetic disorders via whole-exome sequencing of prescreened multiplex consanguineous families. Cell Rep. 10 (2), 148–161. 10.1016/j.celrep.2014.12.015 25558065

[B2] Annunen S. Körkkö J. Czarny M. Warman M. L. Brunner H. G. Kääriäinen H. (1999). Splicing mutations of 54-bp exons in the COL11A1 gene cause Marshall syndrome, but other mutations cause overlapping Marshall/Stickler phenotypes. Am. J. Hum. Genet. 65 (4), 974–983. 10.1086/302585 10486316 PMC1288268

[B3] Bajpai R. Chen D. A. Rada-Iglesias A. Zhang J. Xiong Y. Helms J. (2010). CHD7 cooperates with PBAF to control multipotent neural crest formation. Nature 463 (7283), 958–962. 10.1038/nature08733 20130577 PMC2890258

[B4] Bamshad M. J. Ng S. B. Bigham A. W. Tabor H. K. Emond M. J. Nickerson D. A. (2011). Exome sequencing as a tool for Mendelian disease gene discovery. Nat. Rev. Genet. 12 (11), 745–755. 10.1038/nrg3031 21946919

[B5] Barat‐Houari M. Sarrabay G. Gatinois V. Fabre A. Dumont B. Genevieve D. (2016). Mutation update for COL2A1 gene variants associated with type II collagenopathies. Hum. Pathog. Var. 37 (1), 7–15. 10.1002/humu.22915 26443184

[B6] Basha M. Demeer B. Revencu N. Helaers R. Theys S. Saba S. B. (2018). Whole exome sequencing identifies mutations in 10% of patients with familial non-syndromic cleft lip and/or palate in genes mutated in well-known syndromes. J. Med. Genet. 55 (7), 449–458. 10.1136/jmedgenet-2017-105110 29500247

[B7] Beaty T. H. Marazita M. L. Leslie E. J. (2016). Genetic factors influencing risk to orofacial clefts: today’s challenges and tomorrow’s opportunities. F1000Research 5, 2800. 10.12688/f1000research.9503.1 27990279 PMC5133690

[B8] Berk N. W. Marazita M. L. (2002). Costs of cleft lip and palate: personal and societal implications. Cleft lip palate Orig. Treat. 36, 458–469. “Cleft Lip and Palate” published by Oxford University Press in 2002.

[B9] Bertolacini C. D. P. Ribeiro‐Bicudo L. A. Petrin A. Richieri‐Costa A. Murray J. C. (2012). Clinical findings in patients with GLI2 mutations-phenotypic variability. Clin. Genet. 81 (1), 70–75. 10.1111/j.1399-0004.2010.01606.x 21204792 PMC3135662

[B10] Burnell L. Verchere C. Pugash D. Loock C. Robertson S. Lehman A. (2014). Additional post-natal diagnoses following antenatal diagnosis of isolated cleft lip+/− palate. Arch. Dis. Childhood-Fetal Neonatal Ed. 99 (4), F286–F290. 10.1136/archdischild-2013-305390 24625434

[B11] Cainarca S. Messali S. Ballabio A. Meroni G. (1999). Functional characterization of the opitz syndrome gene product (midin): evidence for homodimerization and association with microtubules throughout the cell cycle. Hum. Mol. Genet. 8 (8), 1387–1396. 10.1093/hmg/8.8.1387 10400985

[B12] Cho H-J. Shin M-Y. Ahn K-M. Lee S. I. Kim H-J. Ki C-S. (2006). X-Linked opitz G/BBB syndrome: identification of a novel mutation and prenatal diagnosis in a Korean family. J. Korean Med. Sci. 21 (5), 790–793. 10.3346/jkms.2006.21.5.790 17043407 PMC2721984

[B13] Chou D. W. Shih C. (2020). Surgical reconstruction of cocaine-induced cleft lip: A case report. Perm. J. 24, 19.197. 10.7812/TPP/19.197 33175674 PMC7213365

[B14] Dao A. M. Goudy S. L. (2016). Cleft palate repair, gingivoperiosteoplasty, and alveolar bone grafting. Facial Plast. Surg. Clin. 24 (4), 467–476. 10.1016/j.fsc.2016.06.005 27712814

[B15] De Falco F. Cainarca S. Andolfi G. Ferrentino R. Berti C. Rodríguez Criado G. (2003). X-Linked opitz syndrome: novel mutations in the MID1 gene and redefinition of the clinical spectrum. Am. J. Med. Genet. Part A 120 (2), 222–228. 10.1002/ajmg.a.10265 12833403

[B16] Elward C. Berg J. Oberlin J. M. Rohena L. (2020). A case series of a mother and two daughters with a GLI2 gene deletion demonstrating variable expressivity and incomplete penetrance. Clin. Case Rep. 8 (11), 2138–2144. 10.1002/ccr3.3085 33235745 PMC7669391

[B17] Figueiredo J. C. Ly S. Magee K. S. Ihenacho U. Baurley J. W. Sanchez‐Lara P. A. (2015). Parental risk factors for oral clefts among central africans, southeast asians, and central Americans. Birth Defects Res. Part A Clin. Mol. Teratol. 103 (10), 863–879. 10.1002/bdra.23417 PMC5049483 26466527

[B18] Fu Z. Yue J. Xue L. Xu Y. Ding Q. Xiao W. (2023). Using whole exome sequencing to identify susceptibility genes associated with nonsyndromic cleft lip with or without cleft palate. Mol. Genet. Genomics 298 (1), 107–118. 10.1007/s00438-022-01967-2 36322204

[B19] Genisca A. E. Frías J. L. Broussard C. S. Honein M. A. Lammer E. J. Moore C. A. (2009). Orofacial clefts in the national birth defects prevention study, 1997–2004. Am. J. Med. Genet. Part A 149 (6), 1149–1158. 10.1002/ajmg.a.32854 PMC3111146 19441124

[B20] Impellizzeri A. Giannantoni I. Polimeni A. Barbato E. Galluccio G. (2019). Epidemiological characteristic of orofacial clefts and its associated congenital anomalies: retrospective study. BMC Oral Health 19 (1), 290–314. 10.1186/s12903-019-0980-5 31870360 PMC6929424

[B21] Jin H. Yingqiu C. Zequn L. Yanjun H. Yunyan Z. Shufan Z. (2018). Chromosomal microarray analysis in the prenatal diagnosis of orofacial clefts: experience from a single medical center in mainland China. Medicine 97 (34), e12057. 10.1097/MD.0000000000012057 30142861 PMC6112896

[B22] Källén K. Robert E. Mastroiacovo P. Castilla E. E. Källén B. (1999). CHARGE association in newborns: A registry‐based study. Teratology 60 (6), 334–343. 10.1002/(SICI)1096-9926(199912)60:6<334:AID-TERA5>3.0.CO;2-S 10590394

[B23] Leslie E. J. Liu H. Carlson J. C. Shaffer J. R. Feingold E. Wehby G. (2016). A genome-wide association study of nonsyndromic cleft palate identifies an etiologic missense variant in GRHL3. Am. J. Hum. Genet. 98 (4), 744–754. 10.1016/j.ajhg.2016.02.014 27018472 PMC4833215

[B24] Li B. Zhou T. Zou Y. (2016). Mid1/Mid2 expression in craniofacial development and a literature review of X‐linked opitz syndrome. Mol. Genet. Genomic Med. 4 (1), 95–105. 10.1002/mgg3.183 26788540 PMC4707030

[B25] Li Y. Y. Tse W. T. Kong C. W. Wong N. K. L. Leung T. Y. Choy K. W. (2022). Prenatal diagnosis and pregnancy outcomes of fetuses with orofacial cleft: A retrospective cohort study in two centres in Hong Kong. Cleft Palate-Craniofac. J., 105566562211284. 10.1177/10556656221128436 36128746

[B26] Luijsterburg A. J. Vermeij-Keers C. (2011). Ten years recording common oral clefts with a new descriptive system. Cleft Palate-Craniofac. J. 48 (2), 173–182. 10.1597/08-150 20500079

[B27] Marcos S. Sarfati J. Leroy C. Fouveaut C. Parent P. Metz C. (2014). The prevalence of CHD7 missense versus truncating mutations is higher in patients with Kallmann syndrome than in typical CHARGE patients. J. Clin. Endocrinol. Metab. 99 (10), E2138–E2143. 10.1210/jc.2014-2110 25077900

[B28] Melkoniemi M. Koillinen H. Männikkö M. Warman M. L. Pihlajamaa T. Kääriäinen H. (2003). Collagen XI sequence variations in nonsyndromic cleft palate, Robin sequence and micrognathia. Eur. J. Hum. Genet. 11 (3), 265–270. 10.1038/sj.ejhg.5200950 12673280

[B29] Panamonta V. Pradubwong S. Panamonta M. Chowchuen B. (2015). Global birth prevalence of orofacial clefts: A systematic review. J. Med. Assoc. Thai 98 (Suppl 7), S11–S21. 26742364

[B30] Park W-J. Theda C. Maestri N. E. Meyers G. A. Fryburg J. S. Dufresne C. (1995). Analysis of phenotypic features and FGFR2 mutations in Apert syndrome. Am. J. Hum. Genet. 57 (2), 321–328. 7668257 PMC1801532

[B31] Reynolds K. Zhang S. Sun B. Garland M. A. Ji Y. Zhou C. J. (2020). Genetics and signaling mechanisms of orofacial clefts. Birth Defects Res. 112 (19), 1588–1634. 10.1002/bdr2.1754 32666711 PMC7883771

[B32] Ribeiro L. A. Murray J. C. Richieri‐Costa A. (2006). PTCH mutations in four Brazilian patients with holoprosencephaly and in one with holoprosencephaly-like features and normal MRI. Am. J. Med. Genet. Part A 140 (23), 2584–2586. 10.1002/ajmg.a.31369 17001668

[B33] Roessler E. Belloni E. Gaudenz K. Jay P. Berta P. Scherer S. W. (1996). Mutations in the human Sonic Hedgehog gene cause holoprosencephaly. Nat. Genet. 14, 357–360. 10.1038/ng1196-357 8896572

[B34] Saleem K. Zaib T. Sun W. Fu S. (2019). Assessment of candidate genes and genetic heterogeneity in human non syndromic orofacial clefts specifically non syndromic cleft lip with or without palate. Heliyon 5 (12), e03019. 10.1016/j.heliyon.2019.e03019 31886431 PMC6921104

[B35] Shkoukani M. A. Chen M. Vong A. (2013). Cleft lip–a comprehensive review. Front. Pediatr. 1, 53. 10.3389/fped.2013.00053 24400297 PMC3873527

[B36] So J. Suckow V. Kijas Z. Kalscheuer V. Moser B. Winter J. (2005). Mild phenotypes in a series of patients with Opitz GBBB syndrome with MID1 mutations. Am. J. Med. Genet. Part A 132 (1), 1–7. 10.1002/ajmg.a.30407 15558842

[B37] Watkins S. E. Meyer R. E. Strauss R. P. Aylsworth A. S. (2014). Classification, epidemiology, and genetics of orofacial clefts. Clin. Plastic Surg. 41 (2), 149–163. 10.1016/j.cps.2013.12.003 24607185

[B38] Wilhelm L. Borgers H. (2010). The ‘equals sign’: A novel marker in the diagnosis of fetal isolated cleft palate. Ultrasound Obstet. Gynecol. 36 (4), 439–444. 10.1002/uog.7704 20521240

[B39] Wilkie A. O. Slaney S. F. Oldridge M. Poole M. D. Ashworth G. J. Hockley A. D. (1995). Apert syndrome results from localized mutations of FGFR2 and is allelic with Crouzon syndrome. Nat. Genet. 9 (2), 165–172. 10.1038/ng0295-165 7719344

[B40] Willie D. Holmes G. Jabs E. W. Wu M. (2022). Cleft palate in Apert syndrome. J. Dev. Biol. 10 (3), 33. 10.3390/jdb10030033 35997397 PMC9397066

[B41] Yu Q. Deng Q. Fu F. Li R. Zhang W. Wan J. (2022). A novel splicing mutation of *ARHGAP29* is associated with nonsyndromic cleft lip with or without cleft palate. J. Maternal-Fetal Neonatal Med. 35 (13), 2499–2506. 10.1080/14767058.2020.1786523 32698641

