# Supplementary material for: Exome sequencing improves genetic diagnosis of congenital orofacial clefts
Source: Front Genet. 2023 Sep 7;14:1252823. doi: 10.3389/fgene.2023.1252823 (PMC10512413; doi:10.3389/fgene.2023.1252823)
Supplement: Supplementary file 1 [file Table1.DOCX]

**Supplementary Table S1** Summary of variants of uncertain significance identified by prenatal exome sequencing in 107 OFCs fetuses

| **Case** | **Ultrasound findings** | **Gene** | **Reference sequence** | **Nucleotide/protein position** | **Mutation type** | **Disease (OMIM ID)** | **Outcome** |
| --- | --- | --- | --- | --- | --- | --- | --- |
| 1 | Cleft palate | *MAF* | NM_005360.4 | c.214A>G  p.(Ser72Gly) | Het, De novo, AD | AGS (601088); CTRCT21, multiple types (610202) | Live birth |
| 2 | Cleft lip | *TTC37* | NM_014639.3 | c.3940G>A p.(Glu1314Lys) | Hom, Mat/Pat, AR | THES1 (222470) | Live birth |
| 3 | Cleft lip | *FRAS1* | NM_025074.7 | c.2835G>A  p.(Gln945=)  c.10041C>G  p.(His3347Gln) | Het, Mat, AR  Het, Pat, AR | FRASRS1 (219000) | Live birth |
| 4 | Cleft lip | *PRR12* | NM_020719.3 | c.404C>T  p.(Ala135Val) | Het, De novo, AD | NOC (619539) | Live birth |
| 5 | Cleft lip and palate | *TWIST1* | NM_000474.3 | c.592A>G  p.(Met198Val) | Het, Pat, AD | CRS1 (123100); Robinow-Sorauf syndrome (180750); SWCOS (617746); SCS with or without eyelid anomalies (101400) | TOP |
| 6 | Cleft lip and palate | *CHD1* | NM_004360.4 | c.1625T>G  p.(Ile542Ser) | Het, Mat, AD | DGLBC with or without cleft lip and/or palate (617682) | Live birth |
| 7 | Cleft lip and palate | *TBX2* | NM_005994.4 | c.1886C>A  p.(Thr629Asn) | Het, De novo, AD | VETD (618223) | Live birth |
| 8 | Cleft lip and palate | *MEGF10* | NM_032446.2 | c.117-5_117-4insC  p.(?)  c.3229G>A  p.(Val1077Ile) | Het, Mat, AR  Het, Pat, AR | CMYP10A (614399); CMYP10B (620249) | TOP |
| 9 | Cleft lip and palate | *DHCR7* | NM_001360.2 | c.289G>A  p.(Ala97Thr)  c.1060G>A  p.(Ala354Thr) | Het, Mat, AR  Het, Pat, AR | SLOS (270400) | TOP |
| 10 | Cleft lip; situs inversus totalis; dextrocardia; double outlet right ventricle; abnormal heart morphology; | *DYNC2H1* | NM_001080463.2 | c.6044G>A  p.(Arg2015Gln)  c.6857C>T  p.(Pro2286Leu) | Het, Mat, AR  Het, Pat, AR | SRTD3 with or without polydactyly (613091) | TOP |
| 11 | Cleft lip and palate; NT 5.8mm; abnormality of the cervical spine; aplasia/Hypoplasia of the lungs | *INTU* | NM_015693.4 | c.544C>A  p.(Leu182Met)  c.1165G>A  p.(Gly389Ser) | Het, Mat, AR  Het, Pat, AR | OFD17 (617926); SRTD20 with polydactyly (617925) | TOP |
| 12 | Cleft lip and palate; micrognathia; single ventricle; pulmonic stenosis; situs inversus totalis | *FLNA* | NM_001110556.2 | c.560A>G  p.(Asn187Ser) | Hemi, Mat,  XL/XLR/XLD | FGS2 (300321); CVDPX (314400); OPD1 (311300) | TOP |
| 13 | Cleft lip; short long bone | *DYNC2H1* | NM_001080463.2 | c.7576A>G  p.(Ile2526Val)  c.7143A>G  p.(Val2381=) | Het, Mat, AR  Het, Pat, AR | SRTD3 with or without polydactyly (613091) | Live birth |
| 14 | Cleft lip and palate; talipes equinovarus; polyhydramnios; abnormality of finger and toe | *SETD2* | NM_014159.6 | c.4537G>A  p.(Glu1513Lys) | Het, De novo, AD | LLS (616831) | TOP |
| 15 | Cleft lip and palate; aplasia/Hypoplasia of the corpus callosum; abnormality of the cardiovascular system | *IGF1R* | NM_000875.5 | c.1552A>G  p.(Arg518Gly) | Het, Mat, AR/AD | IGF1RES (270450) | TOP |
| 16 | Cleft palate; micrognathia | *CUX1* | NM_181552.3 | c.3007C>T  p.(Gln1003Ter) | Het, De novo, AD | GDDI (618330) | TOP |

OFCs: Orofacial clefts; Het: Heterozygous; Hom: Homozygous; Hemi: hemizygous; AD: Autosomal dominant; AR: Autosomal recessive; TOP: Termination of pregnancy; Mat: Maternal inherited; Pat: Paternal inherited; XL, X-linked inheritance; XLR: X-linked recessive; XLD, X-linked dominant inheritance; AGS: Ayme-Gripp Syndrome; CTRCT21: Cataract 21; THES1: Trichohepatoenteric syndrome 1; FRASRS1: Fraser syndrome 1; NOC: Neuroocular syndrome; CRS1: Craniosynostosis 1; SCS: Saethre-Chotzen syndrome; SWCOS: Sweeney-Cox syndrome DGLBC: Diffuse gastric and lobular breast cancer syndrome; VETD: Vertebral anomalies and variable endocrine and T-cell dysfunction; CMYP10A: Congenital myopathy 10A; CMYP10B: Congenital myopathy 10B; SLOS: Smith-Lemli-Opitz syndrome; SRTD3: Short-rib thoracic dysplasia; OFD17: Orofaciodigital syndrome XVII; NT: Nuchal translucency; SRTD20: Short-rib thoracic dysplasia-20; FGS 2: FG syndrome 2; CVDPX: Cardiac valvular dysplasia, X-linked; OPD1: Otopalatodigital syndrome, type I; LLS: Luscan-Lumish syndrom; IGF1RES: Insulin-like growth factor I, resistance to; GDDI: Global developmental delay with or without impaired intellectual development
